# Associations between Diet and Cognitive Function in Stroke Survivors: A Systematic Review and Meta-analysis

**DOI:** 10.1016/j.advnut.2025.100440

**Published:** 2025-05-10

**Authors:** Sasan Amanat, Aimee L Dordevic, Amy Brodtmann, Barbara R Cardoso

**Affiliations:** 1 Department of Nutrition, Dietetics and Food, Monash University, Victoria, Australia; 2 Victorian Heart Institute, Monash University, Victoria, Australia; 3 Department of Neuroscience, School of Translational Medicine, Monash University, Melbourne, Victoria, Australia; 4 Department of Medicine, University of Melbourne, Melbourne, Victoria, Australia; 5 The Florey Institute for Neuroscience and Mental Health, Melbourne, Victoria, Australia

**Keywords:** cognitive impairment, dementia, diet, nutraceuticals, nutrients, nutrition, phytochemicals, stroke

## Abstract

Poststroke cognitive decline is a major form of disability in stroke survivors. Although dietary interventions have shown potential in improving cognitive outcomes in stroke-free populations, their effects on stroke survivors remain unclear. This review aimed to evaluate associations between diet and cognitive function in stroke survivors. MEDLINE, Embase, Scopus, and CINHAL were searched for studies from inception to 16 December, 2024. Eligible articles were observational and interventional studies on adult stroke survivors that evaluated the association/effect of any nutritional exposure/intervention on cognitive performance and dementia risk. Studies were excluded when an intervention was combined with nonnutritional treatment. Random-effects meta-analysis was used for similar randomized clinical trials. This review included 20 clinical trials and 14 observational studies assessing the intake of energy and proteins and a variety of single nutrients, as well as dietary patterns, single foods, and phytochemicals. Meta-analyses revealed a positive effect of energy-protein supplementation on global cognition [standardized mean difference (SMD): 0.62; 95% confidence interval (CI): 0.15, 1.08; *P* = 0.009], and a negative effect of B-vitamins (folic acid, vitamin B6, and vitamin B12) (SMD: −0.40; 95% CI: −0.72, −0.08; *P* = 0.02). Adherence to the Mediterranean-Dietary Approaches to Stop Hypertension (DASH) Intervention for Neurodegenerative Delay and plant-based diets, as well as higher consumption of fruits, milk, coffee, vitamin E, and selenium, were related to better cognitive outcomes; no significant association was observed for adherence to DASH and Mediterranean diets and consumption of vitamins D and C. Butter and sugar intake and calcium supplementation were associated with negative cognitive outcomes. Mixed results were seen for omega (ω)-3, tea, and plant extracts. The available evidence indicates that energy-protein supplementation may benefit cognition after stroke, whereas B-vitamin supplementation has no effect. The substantial heterogeneity among studies hinders conclusions about other dietary strategies.

This review was registered with PROSPERO as CRD42024541785.

**Statements of significance**

This systematic review provides insights into the relationship between diet and poststroke cognitive outcomes, indicating that energy-protein supplementation can enhance the cognitive function of stroke survivors, whereas B-vitamin supplements may negatively impact cognitive outcomes.

## Introduction

Stroke is one of the leading causes of acquired disability and mortality worldwide, affecting over 12 million people each year [1]. Stroke survivors place prevention of poststroke cognitive impairment (PSCI), which can lead to dementia [2], as a high-priority unmet need. About 40% of stroke survivors experience PSCI just 1 y after their stroke [3]. In those who experience major strokes, dementia risk can be up to 50 times higher than that observed in the general population [4]. Cognitive function following a stroke has many trajectories [5]. In the long term, PSCI is influenced by pre-existing disease and cognitive state, including the burden of micro- and macrovascular dysfunction, as well as stroke severity and recurrence [4,[6], [7], [8], [9]].

Currently, there are no effective treatments for PSCI in clinical practice. Preventive strategies such as multidomain interventions that focus on improving modifiable variables, including pharmacological treatment and lifestyle modification, have shown some beneficial effects on poststroke cognition [10]. Although the evidence for the role of diet in poststroke cognitive performance is unclear, dietary components and dietary patterns have shown the potential to improve cognition in high-risk, stroke-free populations. For example, vitamin supplements, particularly folic acid, vitamin C, and vitamin E, have been shown to delay cognitive decline or enhance cognitive function in stroke-free populations [11]. Likewise, a recent systematic review and meta-analysis of randomized controlled trials (RCTs) reported that n–3 PUFAs improved executive function, an indicator of cognitive performance, in middle-aged and older populations, with peak effects at 500 mg/d of n–3 PUFA and 420 mg/d of EPA supplementation [12]. A systematic review and meta-analysis found that high adherence to a Mediterranean diet is associated with a lower risk of mild cognitive impairment and Alzheimer’s disease (AD) [13], whereas low consumption of zinc, selenium, and iron was associated with a higher risk of cognitive impairment in healthy individuals, as well as worse cognitive function in those who already presented with impaired cognition [[14], [15], [16]].

Notably, in the context of stroke patients, preventive strategies may differ from those reported in stroke-free populations. Stroke survivors often face multiple vascular risk factors that raise the risk of developing vascular cognitive impairment [17,18]. Therefore, it may not be appropriate to extrapolate data from nutritional interventions in other populations to stroke survivors. The aim of this systematic review and meta-analysis was to gather the current body of evidence to evaluate the association between diet and cognitive function in stroke survivors.

## Methods

The protocol for this systematic review and meta-analysis was prospectively registered in PROSPERO as CRD42024541785. This review was conducted and reported according to the Cochrane guidelines and the PRISMA statement [19,20].

### Eligibility criteria

Inclusion and exclusion criteria were formed based on the PICOS (population, intervention, comparison, outcome, and study design) format: Population: adults (≥18 y) with a history of any stroke; Intervention: any dietary intervention or exposure, including (but not limited to) dietary patterns, micronutrients, macronutrients, and phytochemicals administered by any route; Comparison: control, placebo, or the lowest percentile of exposure; Outcome: incidence or risk of dementia, incidence or risk of cognitive impairment, cognitive performance; Study design: all observational studies, including prospective and historical cohorts, case-control, and cross-sectional studies, as well as interventional studies (randomized or nonrandomized), were included in this review. Reviews, case studies, or animal studies were excluded. Studies with interventions or exposures that combined dietary elements with other treatments or lifestyle modifications that precluded the assessment of the effect of diet alone were excluded. Included studies should have reported ≥1 of the outcomes of interest on the stroke population. No restrictions on language were applied. Articles published in languages other than English and Spanish were translated in detail using Google Translate. When the required outcomes were not reported, corresponding authors were contacted at least twice via email. The article was excluded if no response was received or the result of interest was unavailable.

### Search strategy

We searched the electronic databases MEDLINE, Scopus, EMBASE, and CINAHL from their inception to 1 May, 2024, and repeated the search on 16 December, 2024. Furthermore, gray literature was searched on Google Scholar (limited to the first 30 records), and the reference lists of the eligible papers were manually scanned to identify eligible papers. The search strategy incorporated a combination of the following terms and Boolean operators: (stroke OR cerebrovascular infarction OR cerebral ischemia) AND (dementia OR cognition OR Alzheimer) AND (nutrition OR diet OR carbohydrate OR amino acid OR lipid OR protein OR fatty acid OR vitamin OR mineral). The complete search strategy is available in Supplemental Table 1.

### Study screening and data extraction

Retrieved articles were imported into Endnote software to identify and eliminate duplicate articles. The remaining articles were then imported into the Covidence web-based tool (Covidence Systematic Review Software, Veritas Health Innovation), which was used to automatically remove duplicates, screen studies, and identify those meeting the prespecified inclusion criteria. After removing duplicates, titles and abstracts were independently screened by 2 of the listed authors (AD, BRC, SA). Full-text screening was conducted for retained articles in a manner similar to the previous screening step. Conflicts were resolved by discussion with a third reviewer.

A single author (SA) conducted the data extraction using a predefined data extraction form, which was subsequently verified by a second author (BRC). Extracted data included article information (title, first author, country in which the study was conducted), study design (study type, intervention/exposure, comparator/control, duration of intervention or follow-up), participant characteristics (time from stroke event to study commencement, number of recruited participants, age, sex), and outcomes (cognitive assessment tools, results of cognitive function, rates of cognitive impairment and dementia).

### Risk of bias assessment

Risk of bias in eligible studies was independently assessed by 2 separate authors (AD, SA) using the Risk of Bias 2 (RoB2) tool [21] for RCTs, Risk of Bias in Non-randomised Studies of Interventions (ROBINS-I) [22] for non-RCTs, cohort, and case-control studies, and the NIH Quality Assessment Tool for Observational Cohort and Cross-sectional Studies [23] for cross-sectional studies. Among the 14 questions in the NIH tool, 3 (Q6, Q7, Q13) do not apply to cross-sectional articles and were therefore not included in the assessment. All conflicts were discussed until a consensus was reached.

### Statistical analysis

Meta-analyses were carried out using Review Manager (RevMan v5.4, Cochrane Collaboration, 2020) for outcomes of interest available in ≥2 RCTs that used similar interventions (B-vitamin and energy-protein supplementations). Standardized mean difference (SMDs) and confidence intervals (CIs) were calculated from mean and SD or odds ratio (OR) (considering the most adjusted regression models) as the effect measure to account for variations in outcome measurements across studies. Mean and SD were estimated using the formula proposed by Wan et al. [24] when outcomes were reported as medians and quartiles. The endpoint values were considered for the B-vitamin meta-analysis, whereas changes from baseline values were considered in the energy and/or protein meta-analysis to address baseline inequalities between study groups. Change SD was imputed using the formula proposed by the Cochrane Handbook [19] for 2 of the studies included in the energy and/or protein meta-analysis. The inverse variance method and random-effects model analysis were used to calculate a pooled effect size. Heterogeneity was assessed using *I*^2^, calculated using RevMan. *I*^2^ thresholds for interpretation of heterogeneity were as follows: 0%–40%: negligible; 30%–60%: may represent moderate heterogeneity; 50%–90%: may represent substantial heterogeneity; and 75%–100%: considerable heterogeneity [19]. Sensitivity analyses were conducted by omitting studies that diverged in terms of intervention; a study that combined B-vitamins with gastrodin supplementation was omitted from the B-vitamins meta-analysis, whereas 2 studies assessing amino acid interventions were omitted from the energy-protein analysis.

## Results

### Study selection

**FIGURE 1:**
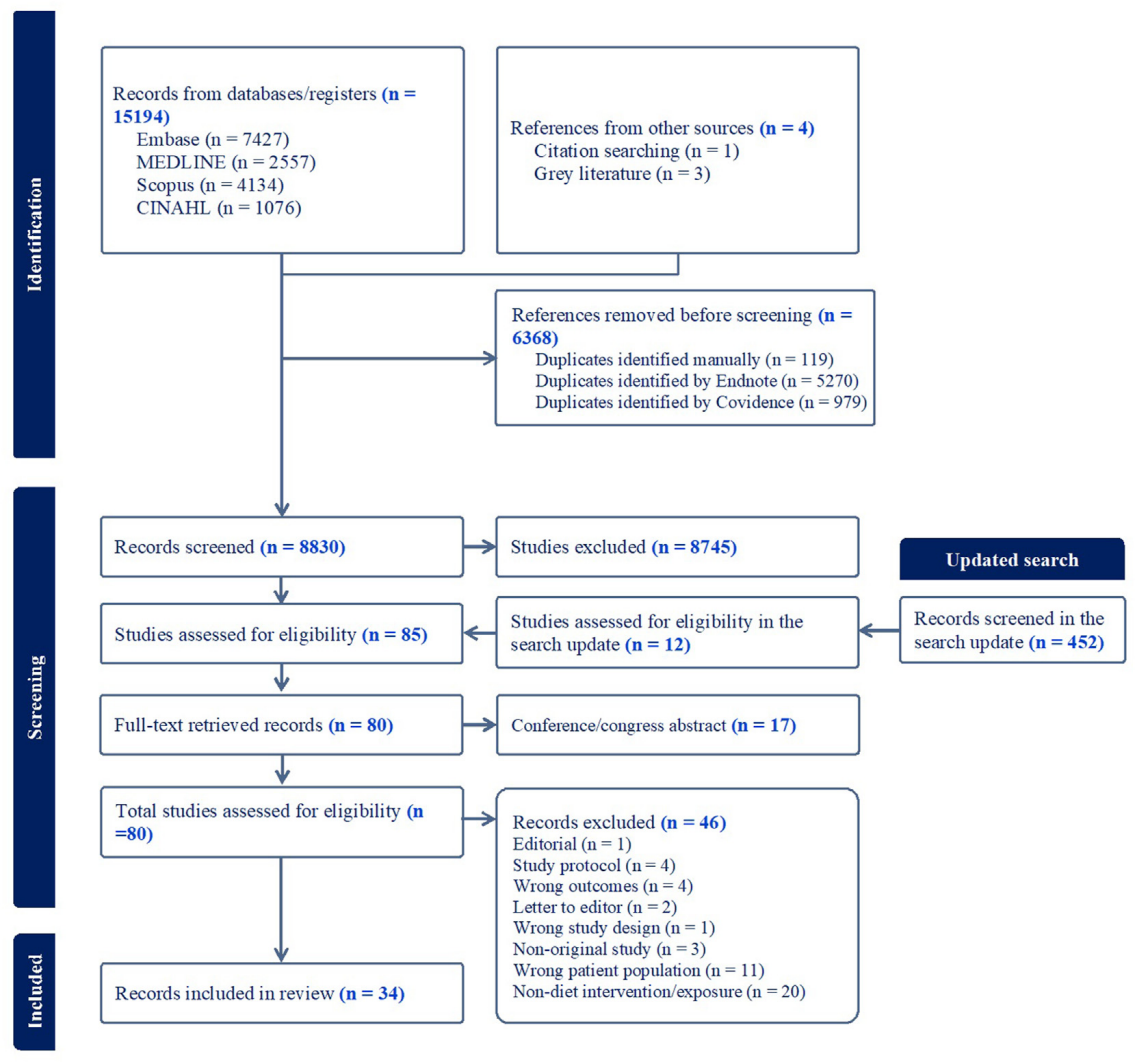
Study selection flow diagram.

The initial search resulted in 15,197 articles, of which 6368 were duplicates. After screening the titles and abstracts, 8745 studies were excluded for not meeting the inclusion criteria and 17 for not providing the full text. Of the remaining 68 full-text articles screened, 36 were excluded primarily because they assessed nondietary interventions/exposures or had the wrong study population. In the search update, the titles and abstracts of 452 and the full texts of 12 articles were screened for eligibility. A total of 34 articles involving 24,849 stroke survivors met the eligibility criteria and were included in this review (Figure 1).

### Study characteristics

Table 1 summarizes the 34 articles included in this review [[25], [26], [27], [28], [29], [30], [31], [32], [33], [34], [35], [36], [37], [38], [39], [40], [41], [42], [43], [44], [45], [46], [47], [48], [49], [50], [51], [52], [53], [54], [55], [56], [57], [58]]. The articles included in this review encompass 16 RCTs [[25], [26], [27], [28], [29], [30],[33], [34], [35], [36], [37], [38], [39], [40],^53^,^55^,^57^,^58^], 2 non-RCTs [54,56], 1 case-control [41], 7 cohort [31,32,44,45,47,52], and 6 cross-sectional [42,43,46,[49], [50], [51]] studies. Four studies [43,44,51,52] assessed large samples represented by a small percentage of stroke survivors, ranging from 3.6% to 15.4%. The studies were conducted across 6 continents and were published between 2004 and 2024. The length of follow-up in clinical trials varied from 1 wk to >7 y. Similarly, the follow-up duration of cohort studies included in this review ranged from 18 d to 10 y.

**TABLE 1:** Dietary intervention/exposure and poststroke cognitive outcomes.

First author (year), country	Study design	Sample size	Age, y, mean (SD)	Sex, % male	Time from stroke to the study commencement	Intervention/exposure	Comparator	Intervention/follow-up duration	Outcomes
Aquilani et al. (2008) [25], Italy	RCT	Intervention: *n* = 24 Control: *n* = 24	Intervention: 73 (6.2) Control: 71 (8.5)	56.25	≥14 d	Energy-protein supplement: 250 kcal of energy, 20 g protein, 28.2 g CHO, and 7 g lipids + normal diet	Normal diet	21 d	↔ MMSE score
Mureşanu et al. (2024) [26], Romania	RCT	Intervention: *n* = 58 Control: *n* = 33	18–80	NR	30–120 d	N-Pep-12: 90 mg/d	None	360 d	**At 90 d:** ↑ MoCA ↔ CTT ↔ DSF ↔DSB ↔ DSC ↔ SS ↔ SSI **At 360 d:** ↑ MoCA ↑ CTT ↔ DSF ↔ DSB ↑ DSC ↑ SS ↔ SSI
Rabadi et al. (2008) [27], United States	RCT	Intervention: *n* = 58 Control: *n* = 58	Intervention: 73.58 (13.02) Control: 75 (10.58)	Intervention: 60 Control: 57	<72 h	240 kcal, 11g protein, 90 mg vitamin C	127 calories, 5 g protein, 36 mg vitamin C	Intervention (mean): 25.98 d Control (mean): 25.44 d	↔ FIM-cognitive score
Otsuki et al. (2020) [28], Japan	RCT	Intervention: *n* = 64 Control: *n* = 64	Intervention median (Q1, Q3) = 78.5 (71, 85) Control median (Q1, Q3) = 80.5 (75, 86)	Intervention: 43.5 Control: 37.5	NR	Intensive energy supply based on the Harris–Benedict equation + stress and activity coefficients	Preadjusted general meals: 25–30 kcal/kg/d	From admission until discharge or ≤3 mo	↔ FIM-cognitive score
Yoshimura et al. (2019) [29], Japan	RCT	Intervention: *n* = 21 Control: *n* = 23	Intervention: 80.8 (7.1) Control: 78.9 (6.3)	Intervention: 10 (33.3) Control: 10 (30.5)	<7 d	Leucine-enriched amino acid supplement	None	8 wk	↔ FIM-cognitive score
Balea et al. (2021) [30], Romania	RCT	Intervention: *n* = 80 Control: *n* = 41	NR	NR	30–120 d	N-Pep-12: 90 mg/d	None	90 d	**At 30 d:** ↔ all tests **At 90 d:** ↑ CTT 1 ↑ SSI ↔ MoCA ↔ CTT 2 ↔ DSF ↔ DSB ↔ DSC ↔ SS
Pellicane et al. (2013) [31], United States	Cohort	55	59.9 (16.3)	53	NR	Protein intake ≥0.8 g/kg/d Energy intake ≥20 kcal/kg/d	Protein intake <0.8 g/kg/d calorie intake <20 kcal/kg/d	Protein intake ≥0.8 g/kg/d (mean): 21.6 d Protein intake <0.8 g/kg/d (mean): 20.3 d Calorie intake ≥20 kcal/kg/d (mean): 18.7 d Calorie intake <20 kcal/kg/d (mean): 21.6 d	↔ FIM-cognitive score
Aquilani et al. (2010) [32], Italy	Cohort	17	75 (8)	58.82	≥14 d	Energy, CHO, protein, and lipid intake	Energy, CHO, protein, and lipid intake	30 d	↑ MMSE score for protein intake ↓ MMSE score for CHO/protein intake
Hankey et al. (2013) [33], 20 countries^1^	RCT	Intervention: *n* = 1110 Control: *n* = 1104	63.3 (11.8)	67.3	<7 mo	B-vitamins: folic acid = 2 mg/d vitamin B6 = 25 mg/d vitamin B12 = 0.5 mg/d	Placebo	Median: 2.8 (1.5–4.6) y	↔ PSCI risk ↔ MMSE score
Tan et al. (2023) [34], Singapore	RCT	Intervention: *n* = 358 Control: *n* = 349	Intervention: 61.5 (11.3) Control: 60.2 (11.5)	Intervention: 64.8 Control: 71.6	<7 mo	B-vitamins: folic acid = 2 mg/d vitamin B6 = 25 mg/d vitamin B12 = 0.5 mg/d	Placebo	≤5 y	↔ Cognitive impairment risk
Almeida et al. (2010) [35], Australia	RCT	Intervention: *n* = 284 Control: *n* = 279	Intervention: 62.9 (12.1) Control: 63.1 (10.5)	Intervention: 66.9 Control: 70.1	<7 mo	B-vitamins: folic acid = 2 mg/d vitamin B6 = 25 mg/d vitamin B12 = 0.5 mg/d	Placebo	Intervention (mean): 7.2 (2.1) y Placebo (mean): 6.9 (2.1) y	↔ Cognitively impaired cases
Andreeva et al. (2011) [36], France	RCT	Group 1: *n* = 117 Group 2: *n* = 95 Group 3: *n* = 100 Placebo: *n* = 100	Group 1: 61.4 (8.7) Group 2: 60.1 (8.7) Group 3: 61.6 (8.8) Placebo: 60.9 (8.9)	NR	NR	Group 1: B-vitamins: folic acid = 0.56 mg/d, vitamin B6 = 3 mg/d, vitamin B12 = 0.02 mg/d Group 2: long-chain ω-3 fatty acids: 600 mg/d EPA and DHA ratio of 2:1 Group 3: B-vitamins and ω-3 fatty acids	Placebo	4 y	↔ F-TICS-m ↔ memory score ↔ recall scores ↑ Temporal orientation score (Group 3 vs. placebo)
Zhou et al. (2017) [37], China	RCT	Intervention: *n* = 46 Control: *n* = 46	Intervention: 58.3 (8.5) Control: 59.1 (7.5)	Intervention: 54.3 Control: 47.8	NR	Gastrodin = 150 mg/d + folic acid = 5 mg/d + vitamin B12 = 75 μg/d + epilepsy medication	Epilepsy medication	6 mo	↓ MoCA score
Toole et al. (2004) [38], United States, Canada, and Scotland	RCT	Intervention: *n* = 1853 Control: *n* = 1827	Intervention: 66.4 (10.8) Control: 66.2 (10.8)	Intervention: 62.2 Control: 62.8	<120 d	High dose B-vitamins (folic acid, vitamin B6, and vitamin B12)	low dose B-vitamins (folic acid, vitamin B6, and vitamin B12)	1 y	↔ MMSE score
Rezaei et al. (2021) [39], Iran	RCT	Intervention: *n* = 30 Control: *n* = 30	Intervention: 62.1 (12.1) Control: 62.6 (10.7)	Intervention: 34.5 Control: 33.3	NR	Vitamin D: single dose = 300,000 IU (IM)	No vitamin D	6 wk	↔ MMSE score
Giovannini et al. (2024) [40], Italy	RCT	Intervention: *n* = 12 Control: *n* = 12	Intervention: 68.9 (14.5) Control: 76.6 (13.9)	Intervention: 58 Control: 50	1–6 mo	SiderAL Med: vitamins (B12, E, C, A, B5, B6, D3, B3, K1, and folate) and minerals (Ca, Mg, Fe, Zn, Se, I, Cu)	None	8 w	**Baseline vs. 16 wk:** ↓ SCWT second ↔ SCWT error ↑ SDMT ↔ TMT ↓ MFIS-Cog **Baseline vs. 4 wk:** ↓ SCWT second ↔ SCWT error ↑ SDMT ↓ TMT ↓ MDIS-Cog **4 wk vs. 8 w,:** ↓ SCWT second ↔ SCWT error ↓ SDMT ↔ TMT ↓ MDIS-Cog **8 wk vs. 16 wk:** ↔ SCWT second ↔ SCWT error ↔ SDMT ↔ TMT ↔ MDIS-Cog
Rabadi et al. (2007) [41], United States	Case-control	Vitamin C: *n* = 23 No vitamin C: *n* = 23	Vitamin C: 76 (11) No vitamin C: 77 (11)	Vitamin C: 56.5 No vitamin C: 56.5	Case: 12 d Control: 11 d	Vitamin C = 1000 mg/d	No vitamin C supplement	12 mo	↔ FIM-cognitive score
Kelleher et al. (2019) [42], United States	Cross-sectional	360	Mean (SEM): 66 (1)	47	Mean (SEM): 9 (1) y	Dietary intake of patients without cognitive impairment: Vitamin C Vitamin D ω-3 PUFAs ω-6 PUFAs Vitamin B6 Folic acid Vitamin B12 Selenium Vitamin E	Dietary intake of patients with cognitive impairment: Vitamin C Vitamin D ω-3 PUFAs ω-6 PUFAs Vitamin B6 Folic acid Vitamin B12 Selenium Vitamin E	NA	Dietary intake of patients without cognitive impairment vs. cognitively impaired: ↔ Vitamin C ↔ Vitamin D ↑ ω-3 PUFAs (g/d) ↑ ω-6 PUFAs (g/d) ↔ Vitamin B6 (mg/d) ↔ Folic acid (μg/d) ↔ Vitamin B12 (μg/d) ↑ Selenium (μg/d) ↑ Vitamin E (mg/d)
Mao et al. (2024) [43], United States	Cross-sectional	159	—	—	NR	CDAI (Q2, Q3, Q4)	CDAI (Q1)	NA	↑ AFT ↑ DSST ↑ Z-score
Kern et al. (2016) [44], Sweden	Cohort	108	Calcium supplement: 80.6 (7.1) No calcium supplement: 75.6 (12.5)	0	NR	Calcium supplement	No calcium supplement	4–6 y	↑ Dementia risk
Cherian et al. (2019) [45], United States	Cohort	106	82.8 (7.1)	27.4	NR	Second and third tertiles for adherence to: MIND diet DASH diet Mediterranean diet	First tertile for adherence to: MIND diet DASH diet Mediterranean diet	Mean: 5.9 y (range: 2–10 y)	↑ Global cognition for MIND diet ↑ Semantic memory for MIND diet ↔ Global cognition for Mediterranean and DASH diet ↔ Semantic memory for Mediterranean and DASH diet
Wang et al. (2022) [46], China	Cross-sectional	83	NR	81.9	At 3 mo	Meat intake Vegetarian diet Mixed diet	—	NA	↔ Cognitively impaired cases
Li et al. (2022) [47], China	Cohort	920	Fish-rich diet: 63.1 (11.7) No fish-rich diet: 62.7 (11.8)	Intervention: 64.5 Control: 66.9	NR	Fish-rich diet ≥5 times/wk	No fish-rich diet	6 y	↓Cognitive impairment risk ↓ Very mild dementia ↑ MMSE score
Akinyemi et al. (2014) [48], Nigeria	Cohort	143	60.4 (9.5)	56.6	At 3 mo	Prestroke daily fish intake	No prestroke daily fish intake	3 m	↓ Cognitive impairment risk
Li et al. (2023) [49], China	Cross-sectional	1047	64.7 (12.7)	64.6	<5 d	Dietary intake of salt, eggs, milk, poultry, pork, beef and mutton, vegetables, fruit, nuts, animal oil, vegetable oil, butter, yogurt	—	NA	↓ Cognitive impairment risk for higher dietary fruit and beef and mutton intake ↑ Cognitive impairment risk for higher dietary butter intake
Tu et al. (2014) [50], China	Cross-sectional	689	68.6 (11.4)	58.6	At 3 mo	Intake of: Fruit Milk Tea Adherence to plant-based diet	—	NA	↓ Cognitive impairment risk for fruit and milk intake↓ Dementia risk for adherence to plant-based diet and drinking tea
Xu et al. (2022) [51], United States	Cross-sectional	2710 (with stroke: 187)	69.1 (0.2)	46.3	NR	High added sugar diet	Normal added sugar diet	NA	↓ Cognitive impairment risk
Zhang et al. (2021) [52], United Kingdom	Cohort	13,352	60.4 (5.1)	45.7	NR	Coffee and/or tea intake	No coffee or tea or no coffee and tea	Median: 7.07 y	↓ Dementia risk in coffee drinkers ↔ Dementia risk in tea drinkers
Li et al. (2022) [53], China	RCT	Intervention: *n* = 64 Control: *n* = 64	Control: 63.8 (7.5) Intervention: 63.9 (7.3)	Intervention: 56.1 Control: 59.6	NR	Modified Guipitang combined with Xuefu Zhuyutang	Red Deer Ginseng	8 wk	↑ MoCA score
Farhana et al. (2016) [54], Indonesia	Quasi-experimental	Intervention 750 mg: *n* = 17 Intervention 1000 mg: *n* = 17 Folic acid: *n* = 14	Intervention 750 mg: 57.3 (10.4) Intervention 1000 mg: 60.3 (11.9) Control: 63.1 (13.2)	Intervention 750 mg: 70.58 Intervention 1000 mg: 52.9 Control: 57.1	After acute phase (not defined)	Gotu kola extract: 750 mg/d 1000 mg/d	Folic acid	6 wk	↔ MoCA score
Bellone et al. (2019) [55], United States	RCT	Intervention: *n* = 8 Control: *n* = 8	Intervention: 58.1 (13.6) Control: 59.6 (13.5)	Intervention: 75 Control: 62.5	2 wk	Pomegranate extract: concentrated blend of polyphenols = 2 g/d	Placebo	1 wk	↔ MMSE v2, ↔ FIM-communication ↔ FIM-social cognition
Belcaro et al. (2024) [56], Italy	Non-RCT	Intervention: *n* = 20 Control: *n* = 18	Intervention: 59.6 (3.1) Control: 58.3 (2)	Intervention: 55 Control: 61.1	4 wk	Pycnogenol = 150 mg/d + health plan	Health plan	6 mo	↑ Simplified cognitive test ↑ Cognitive function item test
Li et al. (2017) [57], China	RCT	Intervention: *n* = 179 Control: *n* = 169	Intervention mean (SEM): 64.5 (0.8) Control mean (SEM): 63.3 (0.8)	Intervention: 32.2 Control: 41.2	≤7 d	Ginkgo biloba extract + aspirin	Aspirin	6 mo	↑ MMSE score ↑ MoCA score
Bonzanino et al. (2024) [58], Italy	RCT	Intervention: *n* = 30 Control: *n* = 30	77.9 (10.3)	55	<72 h	PEALut: palmitoylethanolamide = 1.4 g/d + luteolin = 140 mg/d + thrombolytic therapy	Thrombolytic therapy	90 d	MMSE and MoCA score

Abbreviations: AFT, animal fluency test; CDAI, Composite Dietary Antioxidant Index; CHO, carbohydrate; CTT, Color Trails Test; DASH, Dietary Approaches to Stop Hypertension; DSB, digit span backward test; DSC, digit symbol coding test; DSF, digit span forward test; DSST, digit symbol substitution test; FIM, Functional Independence Measure; F-TICS-m, French version of the modified Telephone Interview for Cognitive Status; IM, intramuscular; MFIS-Cog, Modified Fatigue Impact Scale-cognitive; MIND, Mediterranean-DASH intervention for neurodegenerative delay; MMSE, Mini-Mental State Examination; MoCA, Montreal Cognitive Assessment; NA, not applicable; NR, not reported; PSCI, poststroke cognitive impairment; RCT, randomized controlled trial; SCWT, Stroop color word test; SDMT, symbol digit modalities test; SEM, standard error of the mean; SS, Symbol Search correct; SSI, Symbol Search incorrect; TMT, Trail Making Test.

1 Australia, Austria, Belgium, Brazil, Georgia, Hong Kong, India, Italy, Malaysia, Republic of Moldova, Netherlands, New Zealand, Pakistan, Philippines, Portugal, Serbia, Singapore, Sri Lanka, United Kingdom, United States.

The studies included in this review evaluated the effects or relationships of energy-protein (*n* = 7 [[25], [26], [27], [28], [29], [30], [31], [32]]), B-vitamins (*n* = 5 [[33], [34], [35], [36],^38^]), other micronutrients (*n* = 7 [37,[39], [40], [41], [42], [43], [44]]), omega (ω-3) fatty acids and fish (*n* = 4 [36,42,47,48]), dietary patterns and foods (*n* = 8 [[45], [46], [47], [48], [49], [50], [51], [52]]), and phytochemicals (*n* = 6 [[53], [54], [55], [56], [57], [58]]) on cognitive function, risk of cognitive impairment, and dementia. Cognitive performance was assessed using various tests, with the Mini-Mental State Examination (MMSE) and the Montreal Cognitive Assessment (MoCA) being the most common cognitive tests to evaluate global cognitive function [25,26,30,32,33,[37], [38], [39],[53], [54], [55],^57^,^58^]. In some instances, individual tests were also used to examine specific cognitive domains.

### Risk of bias

**FIGURE 2:**
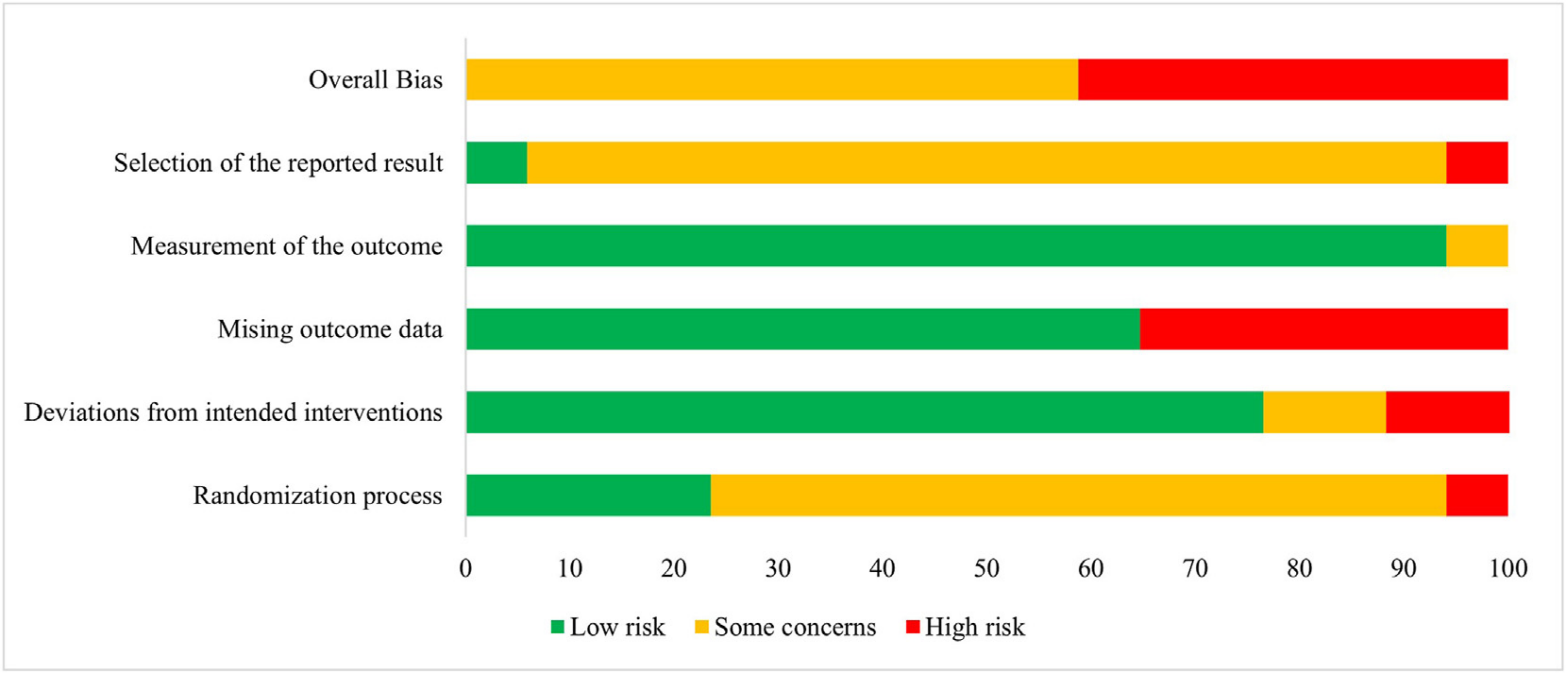
Risk of bias results summary for included randomized controlled trials.

**FIGURE 3:**
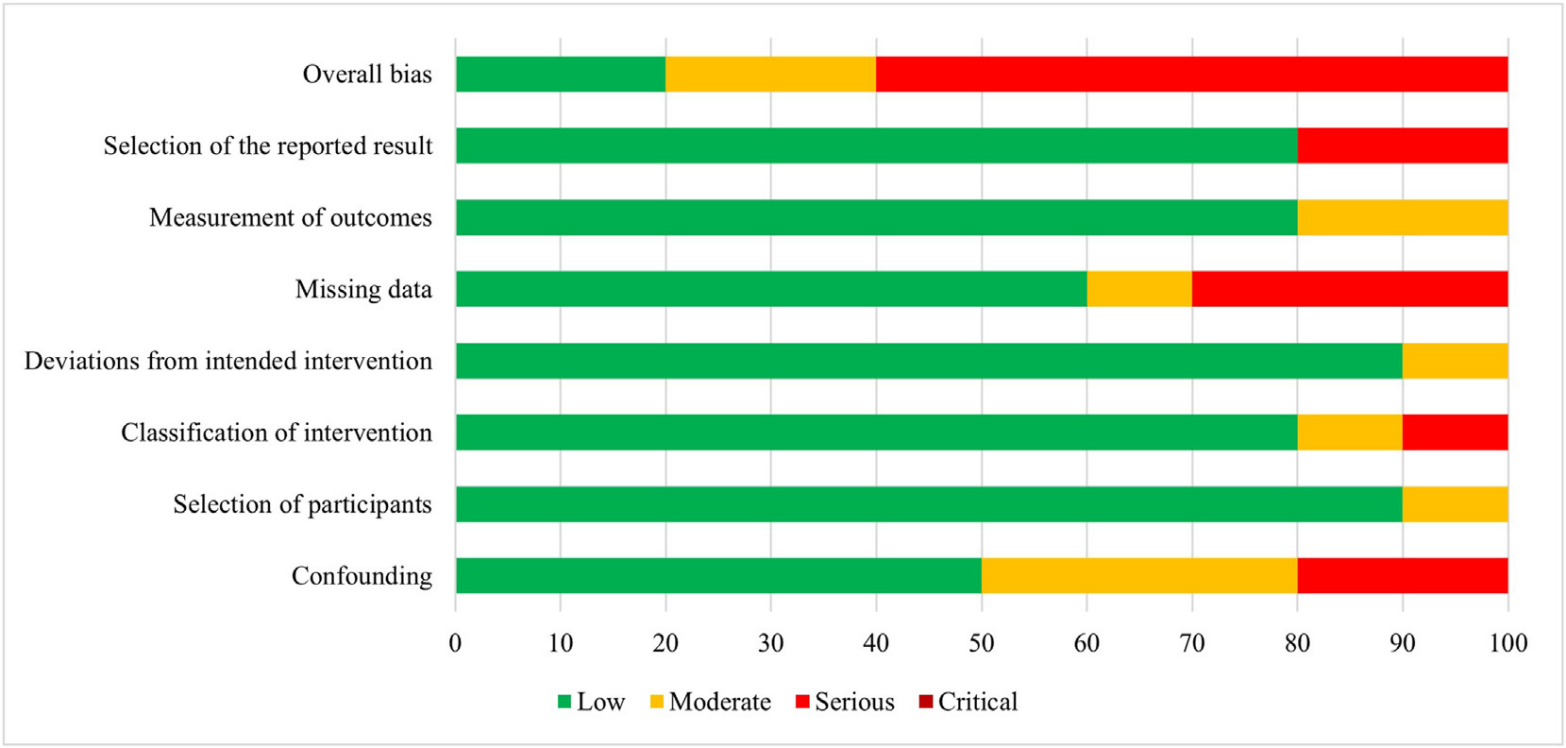
Risk of bias results summary for included nonrandomized trials and cohort and case-control studies.

The assessment of RCTs using the RoB2 tool showed that they had a moderate (48.8%) [25,27,28,30,33,34,[37], [38], [39], [40]] to high (41.2%) [26,29,35,36,53,55,57,58] risk of bias (Figure 2 and Supplemental Figure 1). A common source of bias among these RCTs was the selection of reported results, indicating that almost all studies did not report a prespecified analysis plan. A similar pattern was observed in the randomization process, for which the lack of description of participant allocation concealment raised concerns about the risk of bias. The results from the risk of bias assessment using the ROBINS-I tool indicated that the most common sources of bias were the lack of adjustment for confounders [31,32,41,46,56] and the number of participants lost during follow-up [31,32,44,45], observed in 5 and 4 studies, respectively. Additionally, bias arising from the selection of the reported result [31,48], measurement of outcome [48,56], and classification of intervention [41,46] were detected in 2 studies for each domain (Figure 3 and Supplemental Table 2). The 6 cross-sectional studies [42,43,47,[49], [50], [51]] included in this review were assessed using the NIH tool. These studies mainly had low risk of bias, and although the NIH tool does not provide an overall risk of bias for each study, insufficient reporting was the common source of risk among studies (Supplemental Table 3).

### Energy and proteins

**FIGURE 4:**
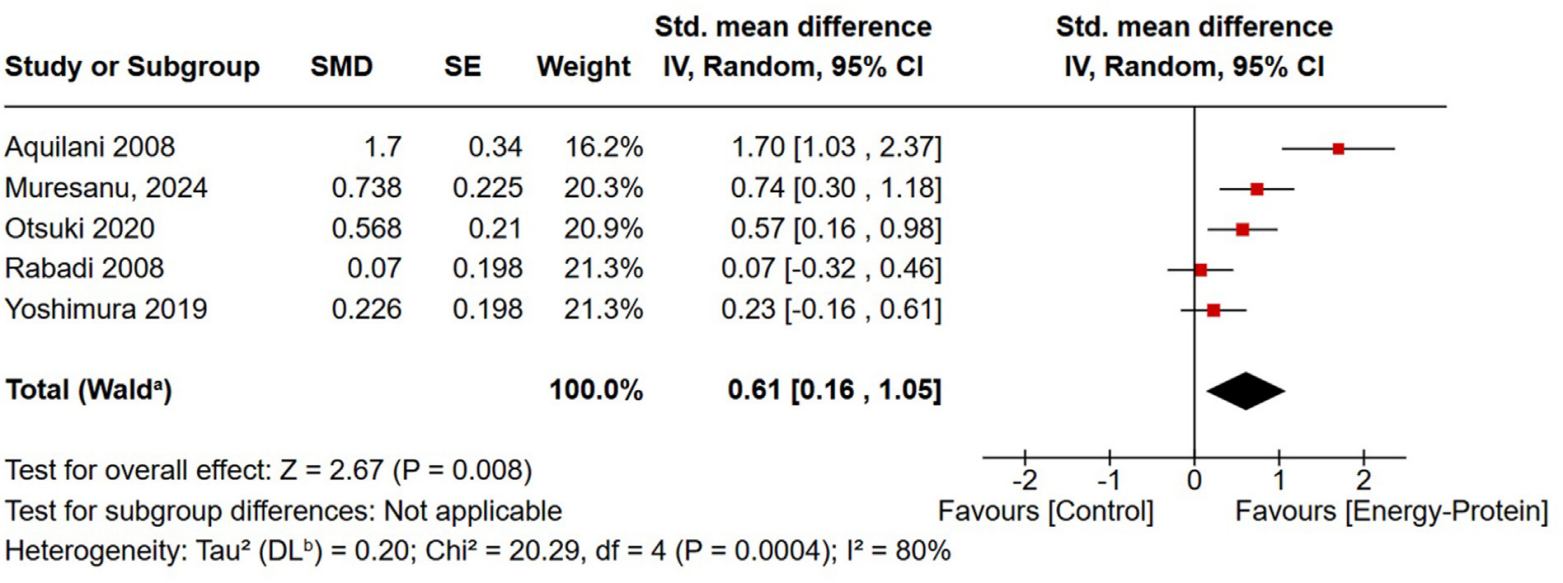
The effect of energy and/or protein supplementation on poststroke global cognitive function. Standardized mean difference (95% CI) shown for individual and pooled trials. CI, confidence interval; DL, DerSimonian-Laird; IV, inverse variance; SE, standard error; SMD, standardized mean difference. a: CI calculated by Wald-type method, b: Tau² calculated by DerSimonian and Laird method.

The results of 5 RCTs [[25], [26], [27], [28], [29]] involving 427 stroke survivors were pooled in the meta-analysis to assess the effects of energy-protein interventions on changes in cognitive performance from baseline (Figure 4). Three RCTs initiated the interventions within the first 2 wk of the stroke event [25,27,29], and 2 articles [26,30] reported on the same participants who started intervention between 30 and 120 d after the stroke event. Although the pilot study [25] reported the results of 90 d of intervention, the full trial that continued for 360 d was included in the analysis [26]. Three RCTs [25,27,28] provided extra daily energy (113–300 kcal) and protein (11–20 g), whereas 1 study [26] supplemented with 90 mg/d mixture of amino acids and peptides (N-Pep-12), and 1 study [29] used 3 g of a leucine-enriched amino acid (40% leucine, 60% other amino acids) supplement daily (Table 1) [[25], [26], [27], [28], [29], [30], [31], [32], [33], [34], [35], [36], [37], [38], [39], [40], [41], [42], [43], [44], [45], [46], [47], [48], [49], [50], [51], [52], [53], [54], [55], [56], [57], [58]]. Although the studies exhibited considerable heterogeneity, the pooled analysis indicated that energy and/or protein supplementation had a significant favorable effect on global cognitive function (SMD: 0.61; 95% CI: 0.16, 1.05) (Figure 4). However, the sensitivity analysis omitting studies with amino acid interventions [26,29] revealed that energy and protein supplementation did not affect cognition (SMD: 0.74; 95% CI: −0.06, 1.54), with high heterogeneity among studies (Supplemental Figure 2).

In a prospective cohort study of 55 patients recently diagnosed with stroke (unclear timeframe), there was no association between cognitive performance [assessed with Functional Independence measure (FIM) cognition subscale] and intake of protein (≥0.8 compared with <0.8 g/kg/d) or energy (≥20 compared with <20 kcal/kg/d) [31]. In contrast, a prospective analysis of 17 patients found that global cognitive function, assessed with MMSE, was positively correlated with protein intake (*r* = 0.65; *P* < 0.001) and negatively correlated with carbohydrate/protein intake (*r* = −0.4; *P* = 0.02), based on aggregated data from baseline (2 wk poststroke) and 30-d follow-up [32].

### B-vitamins

Six articles consisting of 6398 participants addressed the effects of B-vitamins on cognitive performance and PSCI risk in stroke survivors, with intervention durations ranging from 6 mo to >7 y. Five studies tested a daily combination of folic acid (0.56–2.5 mg), vitamin B6 (3–25 mg), and vitamin B12 (20–500μg) [[33], [34], [35], [36],^38^], whereas 1 study examined the effects of folic acid (5 mg) and vitamin B12 (75 μg) combined with the phytochemical gastrodin (150 mg/d) [37]. Three articles [[33], [34], [35]] assessed the same population from the VITATOPS trial, which recruited participants within 7 mo of the stroke event. Since the study populations of these 3 articles overlap, the study with the largest sample size [33] was included in the meta-analysis. Toole et al. [38] included stroke survivors within 120 d after stroke, whereas 2 articles [36,37] did not report the recruitment timeframe relative to stroke occurrence.

**FIGURE 5:**
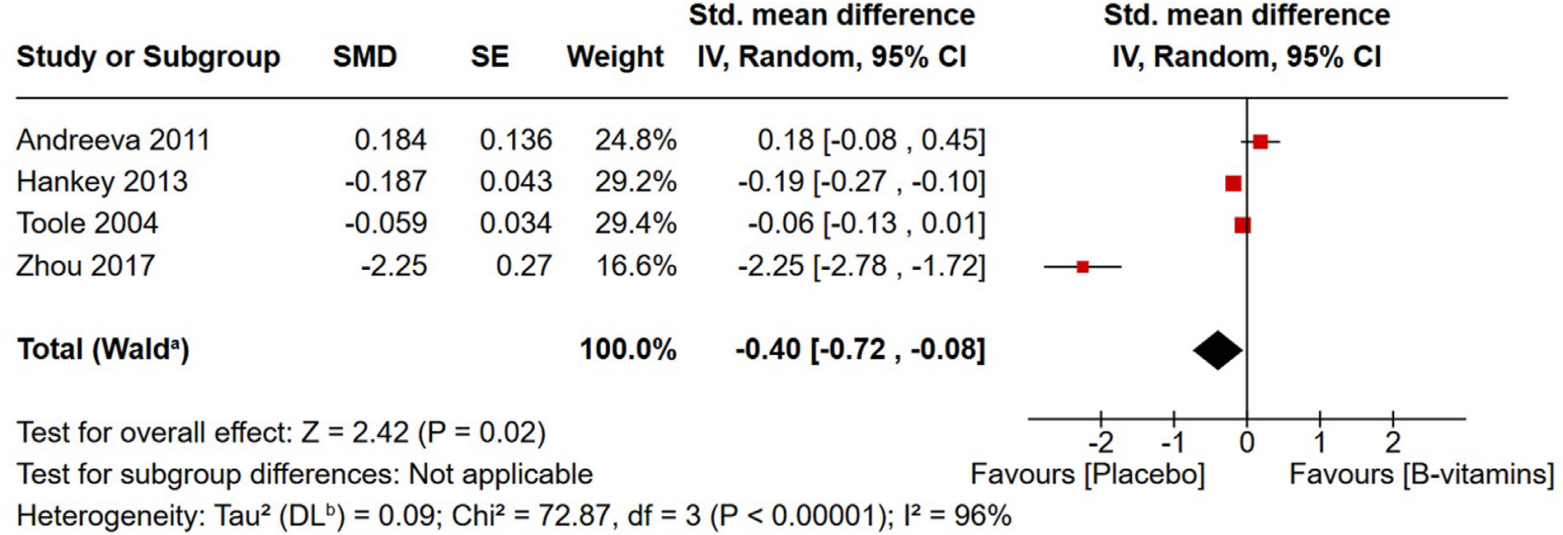
The effect of B-vitamin supplementation on poststroke global cognitive function. Standardized mean difference (95% CI) shown for individual and pooled trials. CI, confidence interval; DL, DerSimonian-Laird; IV, inverse variance; SE, standard error; SMD, standardized mean difference. a: CI calculated by Wald-type method, b: Tau² calculated by DerSimonian and Laird method.

As shown in Figure 5, pooled endpoint data from the 4 studies included in the meta-analysis [33,[36], [37], [38]] showed that B-vitamin supplementation resulted in significantly lower global cognitive function (SMD: −0.40; 95% CI: −0.72, −0.08) compared with controls. In a sensitivity analysis, the omission of the intervention of B-vitamins combined with gastrodin [37] demonstrated that B-vitamin supplementation had no effect on global cognitive function (SMD: −0.07; 95% CI: −0.21, 0.07), although the removal of this study did not reduce the considerable heterogeneity (Supplemental Figure 3).

### Other micronutrients

Six studies (2 RCTs [39,40] and 4 observational studies [[41], [42], [43], [44]]) examined various single or combined micronutrients. One RCT showed that adding a single megadose of intramuscular vitamin D (300,000 IU) to standard care resulted in no benefit in cognitive performance (assessed with MMSE) compared with standard care alone after a 6-wk follow-up period (Δ: 4.67 compared with 3, respectively; *P* = 0.466) [39]. In contrast, stroke survivors receiving a multivitamin and mineral supplement for 8 wk in a pilot study presented less cognitive fatigue (assessed with the Modified Fatigue Impact Scale-cognitive). They performed better in the symbol digit modalities test and Stroop color word test (SCWT) time than the control group at the 16-wk follow-up. However, no differences were observed in the number of errors in the SCWT and Trail Making Test score [40]. A case-control study [41] of 46 recently diagnosed stroke patients showed that changes in FIM-cognitive subtest scores were similar between patients who had a history of daily intake of vitamin C supplement (1000 mg) and controls (with no vitamin C supplementation). Furthermore, a cross-sectional analysis of stroke survivors (*n* = 360) [42] reported that those with and without cognitive impairment had comparable folic acid and vitamins B6, B12, D, and C intakes. In contrast, vitamin E and selenium intakes were significantly higher in the not cognitively impaired group. The Composite Dietary Antioxidant Index, based on the intake of micronutrients with antioxidant properties (manganese, selenium, zinc, and vitamins C, E, and A), was positively associated with performance in the animal fluency test, the digit symbol substitution test, and the composite *z*-score (mean of 3 cognitive tests) in a subsample of stroke survivors (*n* = 159) from the NHANES [43], although no association was observed for the Consortium to Establish a Registry for Alzheimer’s Disease test. Finally, a longitudinal cohort study [44] of 108 dementia-free female stroke survivors found that those with a history of calcium supplement intake were more likely to develop dementia after a 5-y follow-up period (OR: 6.77; 95% CI: 1.36, 33.75).

### ω-3 fatty acids and fish

The association between ω-3 fatty acid intake via supplements or fish and poststroke cognitive outcomes was assessed in 4 studies [36,42,47,48]. The consumption of a fish-rich diet (>5 times/wk) was significantly higher in a group of stroke survivors without dementia (clinical dementia rate [CDR] = 0) when compared with those that were borderline for dementia (CDR = 0.5) [47]. Furthermore, those who consumed a fish-rich diet had a lower risk of developing PSCI (OR: 0.74; 95% CI: 0.46, 0.95) but similar MMSE score compared with the control group (β: 0.13; 95% CI: −0.99, 1.25) in an adjusted model [47]. Akinyemi et al. [48] reported that the odds of developing cognitive impairment 3 mo after stroke was significantly lower in people who consumed fish daily in the year before the stroke event (OR: 0.37; 95% CI: 0.15, 0.89). In a cross-sectional study, Kelleher et al. [42] reported significantly higher intakes of both ω-3 (1.68 g/d compared with 1.36 g/d, *P* < 0.01) and ω-6 (15.12 g/d compared with 12.21 g/d, *P* = 0.04) fatty acids in stroke survivors without cognitive impairment than in those with cognitive impairment. The RCT by Andreeva et al. [36] showed that although 4 y of supplementation with B-vitamins or ω-3 fatty acids (600 mg EPA and DHA in a ratio of 2:1) had no effect on cognitive function, treating stroke survivors with the combination of B-vitamins and ω-3 fatty acid supplements improved their performance in the temporal orientation test compared with placebo (85% compared with 71%, *P* = 0.04).

### Dietary patterns and foods

The association between dietary patterns or food groups and poststroke cognitive performance was examined in 8 observational studies [[45], [46], [47], [48], [49], [50], [51], [52]]. Studies recruited participants at different times after stroke occurrence, from 5 d to ≤6 mo; similarly, the follow-up period of the cohort studies varied from 3 mo to ≤10 y. In a comparison across tertiles of adherence to the Mediterranean-Dietary Approaches to Stop Hypertension (DASH) Intervention for Neurodegenerative Delay (MIND) in 106 stroke survivors, Cherian et al. [45] reported that the MIND diet was positively correlated with higher global cognition (β: 0.083; 95% CI: 0.007, 0.158; *P*-trend = 0.034) and semantic memory (β: 0.070; 95% CI: 0.001, 0.138) after 5.9 y (average) of follow-up. However, no significant associations between adherence to Mediterranean or DASH diets and cognitive outcomes were observed in this population. A cross-sectional study of 83 participants who showed adherence to “meat,” “vegetarian,” and “mixed” dietary patterns 3 mo after stroke was similar between those with and without PSCI, although no clear distinction between these diets was provided [46]. Stroke patients with cognitive impairment (assessed within 5 d of stroke) reported lower intakes of beef, mutton, fruit, nuts, yogurt, poultry, eggs, and milk and higher intakes of salt and butter compared with those without cognitive impairment [49]. Further multivariate logistic regression models revealed that only beef and mutton (OR: 0.80; 95% CI: 0.65,0.98) and fruits (OR: 0.792; 95% CI: 0.67, 0.93) were independently associated with lower risk of acute PSCI, whereas butter intake was associated with higher risk of acute PSCI (OR: 1.44; 95% CI: 1.11, 1.86) [49]. Another cross-sectional study [50] reported that the odds of developing vascular cognitive impairment, no dementia (VCIND) 3 mo after an ischemic stroke was negatively associated with fruit and milk intake (OR: 0.18; 95% CI: 0.09, 0.37 and OR: 0.26, 95% CI: 0.11, 0.61, respectively). In addition, progression to dementia from VCIND was negatively associated with adherence to a plant-based diet (OR: 0.25; 95% CI: 0.10, 0.62) and tea intake (OR: 0.29; 95% CI: 0.09, 0.93). A cross-sectional analysis of United States adults revealed that although stroke is associated with a higher risk of cognitive impairment (OR: 1.59; 95% CI: 1.01, 2.52), having a high amount of added sugar in the diet (highest quartile of added sugar) increases the risk of developing cognitive impairment in stroke survivors (OR: 3.25; 95% CI: 1.09, 9.64) [51]. A cohort study by Zhang et al. [52] found that after a median 7-y follow-up, stroke survivors who drank 0.5 to 3 cups of coffee per day compared to non-coffee drinkers had 21% to 27% lower risk of developing dementia. However, no association with vascular dementia or AD was observed. In addition, consuming ≥4 cups of coffee per day or any amount of tea did not confer any protection against dementia (including vascular dementia and AD), suggesting that the positive effects of coffee may diminish with higher quantities.

### Phytochemicals

The effects of supplementing different nutraceuticals on poststroke cognition were investigated in 6 trials [[53], [54], [55], [56], [57], [58]]. These studies included a total of 602 participants, with intervention durations ranging from 1 wk to 6 mo. Li et al. [53] reported that an 8-wk intervention with a decoction of 15 different plants, combined with additional plant-based decoctions tailored to stroke patients’ symptoms, improved cognitive performance (assessed with MoCA) more effectively than red deer ginseng tablets (2640 mg/d). In a non-RCT, gotu kola extract (750 mg/d or 1000 mg/d) did not show any effect on MoCA scores after 6 wk of intervention when compared with folic acid (3 mg/d) [54]. Furthermore, an RCT found that daily intake of concentrated pomegranate polyphenols (2 g) for 1 wk, starting 2 wk after a stroke event, did not result in significant improvement in MMSE scores or the 2 FIM-cognition subdomains (FIM-communication and FIM-social cognition scores), compared with placebo [55]. In a non-RCT in which participants consumed 150 mg/d Pycnogenol (maritime pine bark extract) for 6 mo, the stroke patients showed better performance in several cognitive tasks assessing global cognitive function, attention, memory, and executive function compared with a control group receiving no intervention [56]. When ischemic stroke patients received a daily dose of Ginkgo biloba extract (450 mg) plus 100 mg aspirin for 6 mo, they experienced significantly less decline in cognitive performance (assessed with MMSE and MoCA) over a period of 180 d compared with a control group (100 mg/d aspirin) [57]. In an RCT of acute (within 72 h) stroke patients, 30 participants receiving 10 mL of PEALut supplement (luteolin + palmitoylethanolamide) twice a day for 90 d experienced improvement in MMSE and MoCA scores from the baseline measures. However, given the limited number of participants in the control group who completed the cognitive tests, comparisons between the 2 groups were not performed [58].

## Discussion

We found 34 studies that examined macro- and micronutrients, foods, phytochemicals, and dietary patterns and poststroke cognitive status. Variations in their design, outcome measurement tools, and, most importantly, the interventions or exposures, posed a challenge in forming a cohesive understanding of the link between diet and cognitive outcomes in stroke survivors. The main findings indicate that although supplementing stroke patients with energy-protein and amino acids starting in the acute and subacute phases of stroke appears to have the potential to enhance cognitive function, B-vitamin supplementation may not affect poststroke outcomes in cognition.

Stroke survivors may present with difficulty meeting their nutritional needs due to dysphagia, restricted movement, visuospatial impairment, and depression [[59], [60], [61]]. Insufficient energy consumption leads to a negative energy balance, contributing to higher mortality rates, prolonged hospital stays, and poorer rehabilitation outcomes [62]. Thus, research has focused on using energy and protein-rich supplements to minimize the gap between energy expenditure and consumption. Although the pooled results of energy-protein plus amino acid supplementation showed a positive effect on cognitive function, a sensitivity analysis excluding studies with amino acid interventions suggests that these amino acids may be important for the effectiveness of energy-protein supplementation. Leucine, along with other branched-chain amino acids (BCAAs), serves as a metabolic precursor for neurotransmitter synthesis and provides an important nitrogen source to support the production of glutamate and glutamine, essential brain metabolites. The critical role of BCAAs has also been shown to aid recovery from brain-related conditions such as traumatic brain injury in animal models [63]. Furthermore, N-Pep-12, a peptide produced enzymatically from purified nerve cell proteins, has also shown neuroprotective effects in healthy older adults, with proposed effects on antiapoptotic factors and enhancing neuron resilience in metabolic disturbance [64,65].

B-vitamins, particularly folic acid, vitamin B12, and vitamin B6, are essential cofactors of enzymes responsible for homocysteine clearance, a target metabolite related to an increased risk of AD and cardiovascular disease [66,67]. The efficacy of B-vitamin supplementation in preventing stroke or cognitive decline through homocysteine clearance has been reported previously [68,69]; however, our pooled analysis found no benefit of B-vitamin supplementation on poststroke cognitive performance and suggested a potential adverse effect when combined with gastrodin. The null findings may be partially explained by the adequate plasma concentrations of homocysteine observed in most of the supplemented groups. Given that the effect of B-vitamins in improving neurovascular damage is hypothesized to occur via the reduction of homocysteine, it is plausible that they would not provide additional benefit to patients with normal homocysteine levels [70]. In addition, Andreeva et al. [36] suggested that combining B-vitamins with ω-3 fatty acid supplementation positively affects poststroke cognition, corroborating findings from stroke-free populations [71]. One possible explanation is that B-vitamins can enhance the transport of ω-3 fatty acids to the brain by accelerating the conversion of phosphatidylcholine, high in ω-3 fatty acids, from phosphatidylethanolamine [72]. This aligns with a report from Schaefer et al. [73] that a high level of plasma phosphatidylcholine-DHA is related to a lower risk of all-cause dementia.

Adherence to healthy diets has been associated with cognitive improvements in stroke-free middle-aged and older populations [74,75]. Nonetheless, data from stroke patients is very limited, with only 1 study assessing the most investigated dietary patterns (MIND, Mediterranean, and DASH diets) in cognitive decline and dementia [45]. In that study, the MIND diet was more promising in preventing poststroke cognitive decline than the DASH and Mediterranean diets, possibly due to its emphasis on foods related to brain health, such as berries and leafy green vegetables, rather than a primary focus on cardiovascular health. Additionally, studies investigating the association between the consumption of antioxidant-rich foods [43,49,50,52] shed light on the potential benefits of increasing the consumption of nutrients and foods with antioxidant profiles to enhance poststroke cognitive performance.

We have expanded our research to encompass studies that assessed the effects of nutraceuticals on poststroke cognitive status because these compounds are derived from foods. In that regard, we identified studies examining compounds that have been previously tested in stroke-free populations, such as pomegranate, Ginkgo biloba and gotu kola extracts, pycnogenol, and modified Guipitang combined with Xuefu Zhuyutang [[76], [77], [78], [79], [80], [81]]. Nonetheless, we noted that the studies examining the effects of phytochemicals and plant extracts on poststroke cognitive performance were all of low quality, with small sample sizes and short intervention periods, hindering clear evidence.

This review is the first to synthesize current evidence on the association of diet with poststroke cognitive status. However, the limitations are worth mentioning. The number of studies evaluating similar dietary interventions was limited, hindering comparisons and synthesis of findings for most interventions. Furthermore, the lack of dietary intake data in the intervention studies is a significant limitation, as it prevents the assessment of potential interactions between diet quality and other nutritional factors with the intervention, which may influence participant outcomes. In addition, supplementation to nutrient-replete populations may have no additional benefits or even cause adverse effects [82], and thus, nutritional status before supplementation should be considered. Cognitive status was usually part of the secondary outcomes in the studies, and thus, several studies were underpowered to detect differences in cognition related to diet. Furthermore, the variability of tools used to assess cognitive status also made comparing findings across the studies difficult; for example, the diagnosis of dementia or cognitive impairment was established using different measures and cutoffs. We also note that interventions and assessments were conducted in different poststroke recovery phases (acute, subacute, and chronic) across the studies. Although stroke survivors can experience different trajectories in cognitive function over time, cognition is typically marked by a sudden decline after stroke onset, partial recovery within 3 to 6 mo, and a more pronounced gradual decrease in the chronic phase compared to nonstroke individuals with similar risk factors [9]. Therefore, different recovery phases may require distinct dietary considerations for optimal outcomes, and comparing studies assessing different timeframes may be misleading.

### Conclusions

In conclusion, this systematic review and meta-analysis suggests that energy-protein and amino acid supplementation initiated during the acute and subacute phases of stroke may support cognitive improvement, whereas B-vitamin supplementation appears to have no effect on poststroke cognitive outcomes. The considerable variation in study methodologies across studies highlights the need for further high-quality trials investigating the impact of dietary strategies to improve cognition in stroke survivors.

## Author contributions

The authors’ responsibilities were as follows – SA, BRC, ALD, AB: designed the research; SA, BRC, ALD: conducted the research; SA, BRC, ALD: prepared the data; SA: synthesized the data; SA, BRC, ALD: wrote the article with editorial assistance from AB; and all authors: primary responsibility for the final content and read and approved the final manuscript.

## Data availability

Data described in the manuscript, code book, and analytic code will be made publicly and freely available without restriction in the supplementary data.

## Funding

The authors reported no funding received for this study.

## Conflict of interest

The authors report no conflicts of interest.
