# Supplementary material for: Associations between Diet and Cognitive Function in Stroke Survivors: A Systematic Review and Meta-analysis
Source: Adv Nutr. 2025 May 10;16(6):100440. doi: 10.1016/j.advnut.2025.100440 (PMC12164037; doi:10.1016/j.advnut.2025.100440)
Supplement: multimedia component 1 [file mmc1.docx]

**Associations between diet and cognitive function in stroke survivors: A systematic review and meta-analysis**

Sasan Amanat, Aimee L Dordevic, Amy Brodtmann, Barbara R Cardoso

**Supplementary Table 1:** Search strategies for databases MEDLINE, Scopus, EMBASE, and CINAHL databases.

| MEDLINE (Ovid):  ((stroke or "brain Infarction" or (cerebrovascular adj1 (event* or onset* or accident*)) or CVA or ((brain or h?emorrhagic or ischemi* or cerebral) adj1 infarct*) or ((cerebral or brain or Intracerebral or Intracranial or Subarachnoid) adj (infarct or Thrombosis or emboli or ischemia)) or poststroke or post-stroke).tw. or "brain infarction"/ or stroke/) and ((dementia or Cogniti* or neurocognitive or (memory adj (disorder* or dysfunction or decline or decrease or impairment)) or Alzheimer).tw. or dementia/ or "cognitive disorders"/ or "alzheimer disease"/) and (carbohydrate/ or "amino acids"/ or exp vitamins/ or diet.tw. or lipids/ or lipid*.tw. or carbohydrate*.tw. or protein*.tw. or "amino acid*".tw. or "fatty acid*".tw. or supplement*.tw. or nutraceutical.tw. or vitamin*.tw. or mineral*.tw. or malnutrition/ or nutrition*.tw. or nutrient*.tw. or diet/ or "trace elements"/ or " dietary Proteins"/) |
| --- |
| EMBASE (Ovid):  ((stroke.tw.) OR ("brain Infarction".tw.) OR ((cerebrovascular ADJ1 (event* OR onset* OR accident*)).tw.) OR (CVA.tw.) OR (((brain OR h?emorrhagic OR ischemi* OR cerebral) ADJ1 infarct*).tw.) OR (((cerebral OR brain OR intracerebral OR intracranial OR subarachnoid) ADJ (infarct OR thrombosis OR emboli OR ischemia)).tw.) OR (poststroke.tw.) OR (post-stroke.tw.) OR ("brain infarction"/) OR ("cerebrovascular accident"/)) AND ((dementia.tw.) OR (cogniti*.tw.) OR (neurocognitive.tw.) OR ((memory ADJ (disorder* OR dysfunction OR decline OR decrease OR impairment)).tw.) OR (alzheimer.tw.) OR (dementia/) OR ("cognitive defect"/) OR ("alzheimer disease"/)) AND ((carbohydrate/) OR ("amino acid"/) OR (exp vitamin/) OR (diet.tw.) OR (lipid/) OR (lipid*.tw.) OR (carbohydrate*.tw.) OR (protein*.tw.) OR ("amino acid*".tw.) OR ("fatty acid*".tw.) OR (supplement*.tw.) OR (nutraceutical.tw.) OR (vitamin*.tw.) OR (mineral*.tw.) OR (malnutrition/) OR (nutrition*.tw.) OR (nutrient*.tw.) OR (diet/) OR ("trace element"/) OR ("protein diet"/)) |
| Scopus:  ( ( TITLE-ABS ( stroke ) ) OR ( TITLE-ABS ( "brain Infarction" ) ) OR ( TITLE-ABS ( cerebrovascular W/1 ( event* OR onset* OR accident* ) ) ) OR ( TITLE-ABS ( cva ) ) OR ( TITLE-ABS ( ( brain OR haemorrhagic OR hemorrhagic OR ischemi* OR cerebral ) W/1 infarct* ) ) OR ( TITLE-ABS ( ( cerebral OR brain OR intracerebral OR intracranial OR subarachnoid ) W/1 ( infarct OR thrombosis OR emboli OR ischemia ) ) ) OR ( TITLE-ABS ( poststroke ) ) OR ( TITLE-ABS ( post-stroke ) ) OR ( INDEXTERMS ( "brain infarction" ) ) OR ( INDEXTERMS ( "cerebrovascular accident" ) ) ) AND ( ( TITLE-ABS ( dementia ) ) OR ( TITLE-ABS ( cogniti* ) ) OR ( TITLE-ABS ( neurocognitive ) ) OR ( TITLE-ABS ( memory W/1 ( disorder* OR dysfunction OR decline OR decrease OR impairment ) ) ) OR ( TITLE-ABS ( alzheimer ) ) OR ( INDEXTERMS ( dementia ) ) OR ( INDEXTERMS ( "cognitive defect" ) ) OR ( INDEXTERMS ( "Alzheimer disease" ) ) ) AND ( ( INDEXTERMS ( carbohydrate ) ) OR ( INDEXTERMS ( "amino acid" ) ) OR ( INDEXTERMS ( vitamin ) ) OR ( TITLE-ABS ( diet ) ) OR ( INDEXTERMS ( lipid ) ) OR ( TITLE-ABS ( lipid* ) ) OR ( TITLE-ABS ( carbohydrate* ) ) OR ( TITLE-ABS ( protein* ) ) OR ( TITLE-ABS ( "amino acid*" ) ) OR ( TITLE-ABS ( "fatty acid*" ) ) OR ( TITLE-ABS ( supplement* ) ) OR ( TITLE-ABS ( nutraceutical ) ) OR ( TITLE-ABS ( vitamin* ) ) OR ( TITLE-ABS ( mineral* ) ) OR ( INDEXTERMS ( malnutrition ) ) OR ( TITLE-ABS ( nutrition* ) ) OR ( TITLE-ABS ( nutrient* ) ) OR ( INDEXTERMS ( diet ) ) OR ( INDEXTERMS ( "trace element" ) ) OR ( INDEXTERMS ( "protein diet" ) ) ) AND ( EXCLUDE ( DOCTYPE , "re" ) ) AND ( LIMIT-TO ( EXACTKEYWORD , "Human" ) OR LIMIT-TO ( EXACTKEYWORD , "Humans" ) ) |
| CINAHL:  (((TI stroke OR AB stroke)) OR ((TI "Brain Infarction" OR AB "Brain Infarction")) OR (((TI Cerebrovascular OR AB Cerebrovascular) N1 ((TI event* OR AB event*) OR (TI onset* OR AB onset*) OR (TI Accident* OR AB Accident*)))) OR ((TI CVA OR AB CVA)) OR ((((TI brain OR AB brain) OR (TI H#emorrhagic OR AB H#emorrhagic) OR (TI Ischemi* OR AB Ischemi*) OR (TI cerebral OR AB cerebral)) N1 (TI infarct* OR AB infarct*))) OR ((((TI cerebral OR AB cerebral) OR (TI brain OR AB brain) OR (TI Intracerebral OR AB Intracerebral) OR (TI Intracranial OR AB Intracranial) OR (TI Subarachnoid OR AB Subarachnoid)) W1 ((TI infarct OR AB infarct) OR (TI Thrombosis OR AB Thrombosis) OR (TI Emboli OR AB Emboli) OR (TI ischemia OR AB ischemia)))) OR ((TI Poststroke OR AB Poststroke)) OR ((TI Post-stroke OR AB Post-stroke)) OR ((MH "stroke"))) AND (((TI dementia OR AB dementia)) OR ((TI Cogniti* OR AB Cogniti*)) OR ((TI Neurocognitive OR AB Neurocognitive)) OR (((TI memory OR AB memory) W1 ((TI disorder* OR AB disorder*) OR (TI dysfunction OR AB dysfunction) OR (TI decline OR AB decline) OR (TI decrease OR AB decrease) OR (TI impairment OR AB impairment)))) OR ((TI Alzheimer OR AB Alzheimer)) OR ((MH dementia)) OR ((MH "Alzheimer disease"))) AND (((MH carbohydrates)) OR ((MH "amino acids")) OR ((MH vitamins+)) OR ((TI diet OR AB diet)) OR ((MH lipids)) OR ((TI lipid* OR AB lipid*)) OR ((TI carbohydrate* OR AB carbohydrate*)) OR ((TI protein* OR AB protein*)) OR ((TI "amino acid*" OR AB "amino acid*")) OR ((TI "fatty acid*" OR AB "fatty acid*")) OR ((TI supplement* OR AB supplement*)) OR ((TI nutraceutical OR AB nutraceutical)) OR ((TI vitamin* OR AB vitamin*)) OR ((TI mineral* OR AB mineral*)) OR ((MH malnutrition)) OR ((TI nutrition* OR AB nutrition*)) OR ((TI nutrient* OR AB nutrient*)) OR ((MH diet)) OR ((MH "trace elements")) OR ((MH " dietary Proteins "))) |

| **Study ID** | **D1** | **D2** | **D3** | **D4** | **D5** | **Overall** |  |  |  |
| --- | --- | --- | --- | --- | --- | --- | --- | --- | --- |
| Hankey, 2013 |  |  |  |  |  |  |  |  | Low risk |
| Tan, 2023 |  |  |  |  |  |  |  |  | Some concerns |
| Almeida, 2010 |  |  |  |  |  |  |  |  | High risk |
| Li, W. H, 2022 |  |  |  |  |  |  |  |  |  |
| Andreeva, 2011 |  |  |  |  |  |  |  | D1 | Randomisation process |
| Rezaei, 2021 |  |  |  |  |  |  |  | D2 | Deviations from the intended interventions |
| Aquilani, 2008 |  |  |  |  |  |  |  | D3 | Missing outcome data |
| Balea, 2021 |  |  |  |  |  |  |  | D4 | Measurement of the outcome |
| Otsuki, 2020 |  |  |  |  |  |  |  | D5 | Selection of the reported result |
| Rabadi, 2008 |  |  |  |  |  |  |  |  |  |
| Bellone, 2019 |  |  |  |  |  |  |  |  |  |
| Zhou, 2017 |  |  |  |  |  |  |  |  |  |
| Bonzanino, 2024 |  |  |  |  |  |  |  |  |  |
| Li, 2017 |  |  |  |  |  |  |  |  |  |
| Toole, 2004 |  |  |  |  |  |  |  |  |  |
| Yoshimura, 2019 |  |  |  |  |  |  |  |  |  |
| Muresanu, 2024 |  |  |  |  |  |  |  |  |  |
| Giovannini, 2024 |  |  |  |  |  |  |  |  |  |

**Supplementary Figure 1:** Risk of bias of included RCT studies assessed with ROB2 tool.

| Study ID | Confounding | Selection of participants | Classification of intervention | Deviations from intended intervention | Missing data | Measurement of outcomes | Selection of the reported result | overall |
| --- | --- | --- | --- | --- | --- | --- | --- | --- |
| Belcardo, 2024 | Serious | Low | Low | Low | Low | Moderate | Low | Serious |
| Kern, 2016 | Low | Low | Low | Moderate | Serious | Low | Low | Serious |
| Farhana, 2016 | Low | Low | Low | Low | Low | Low | Low | Low |
| Zhang, 2021 | Low | Low | Low | Low | Low | Low | Low | Low |
| Pellicane, 2013 | Moderate | Low | Low | Low | Serious | Low | Serious | Serious |
| Akinyemi, 2014 | Low | Low | Low | Low | Low | Moderate | Serious | Serious |
| Cherian, 2019 | Low | Low | Low | Low | Moderate | Low | Low | Moderate |
| Wang, 2022 | Serious | Low | Serious | Low | Low | Low | Low | Serious |
| Rabadi, 2007 | Moderate | Low | Moderate | Low | Low | Low | Low | Moderate |
| Aquilani, 2010 | Moderate | Moderate | Low | Low | Serious | Low | Low | Serious |

**Supplementary Table 2:** Risk of bias of included non-randomised trials, cohort and case-control studies assessed via ROBINS-I tool.

| Study ID | Q1: Was the research question or objective in this paper clearly stated? | Q2: Was the study population clearly specified and defined? | Q3: Was the participation rate of eligible persons at least 50%? | Q4: Were all the subjects selected or recruited from the same or similar populations (including the same time period)? Were inclusion and exclusion criteria for being in the study prespecified and applied uniformly to all participants? | Q5: Was a sample size justification, power description, or variance and effect estimates provided? | Q6: For the analyses in this paper, were the exposure(s) of interest measured prior to the outcome(s) being measured? | Q7: Was the timeframe sufficient so that one could reasonably expect to see an association between exposure and outcome if it existed? | Q8: For exposures that can vary in amount or level, did the study examine different levels of the exposure as related to the outcome (e.g., categories of exposure, or exposure measured as continuous variable)? | Q9: Were the exposure measures (independent variables) clearly defined, valid, reliable, and implemented consistently across all study participants? | Q10: Was the exposure(s) assessed more than once over time? | Q11: Were the outcome measures (dependent variables) clearly defined, valid, reliable, and implemented consistently across all study participants? | Q12: Were the outcome assessors blinded to the exposure status of participants? | Q13: Was loss to follow-up after baseline 20% or less? | Q14: Were key potential confounding variables measured and adjusted statistically for their impact on the relationship between exposure(s) and outcome(s)? |
| --- | --- | --- | --- | --- | --- | --- | --- | --- | --- | --- | --- | --- | --- | --- |
| Mao, 2024 | Yes | Yes | Yes | Yes | NR | No | No | Yes | Yes | Yes | Yes | NR | NA | Yes |
| Li, 2022 | Yes | Yes | No | Yes | NR | No | No | Yes | Yes | Yes | Yes | NR | NA | Yes |
| Li, 2023 | Yes | Yes | NR | Yes | NR | No | No | No | Yes | No | Yes | NR | NA | Yes |
| Tu, 2014 | No | Yes | YES | Yes | Yes | No | No | No | NR | No | Yes | NR | NA | Yes |
| Xu, 2022 | Yes | Yes | Yes | Yes | NR | No | No | Yes | Yes | NR | Yes | NR | NA | Yes |
| Kelleher, 2019 | Yes | Yes | Yes | Yes | NR | No | No | Yes | Yes | No | Yes | NR | NA | Yes |

**Supplementary Table 3:** Risk of bias of included cross-sectional studies assessed with NIH quality assessment tool. NR: not reported, NA: not applicable.


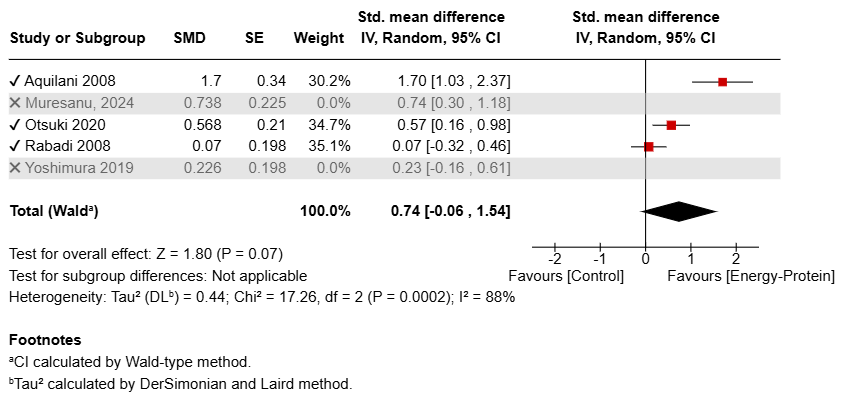


**Supplementary Figure 2:** Sensitivity analysis: Effects of energy-protein interventions (excluding amino acid interventions) on the cognitive function of stroke survivors. Standardised mean difference (95% CI) shown for individual and pooled trials.


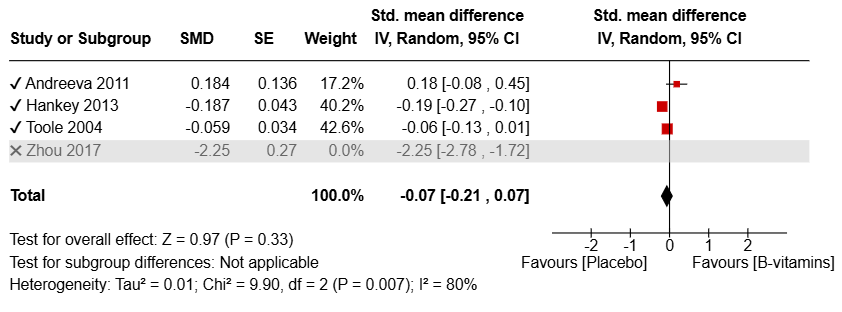


**Supplementary Figure 3:** Sensitivity analysis: Effects of B-vitamins interventions on cognitive outcomes of stroke survivors (excluding gastrodin intervention). Standardised mean difference (95% CI) shown for individual and pooled trials.
